# Active Commuting and Depression Symptoms in Adults: A Systematic Review

**DOI:** 10.3390/ijerph17031041

**Published:** 2020-02-06

**Authors:** Adilson Marques, Miguel Peralta, Duarte Henriques-Neto, Diana Frasquilho, Élvio Rubio Gouveira, Diego Gomez-Baya

**Affiliations:** 1 CIPER, Faculdade de Motricidade Humana, Universidade de Lisboa, Estrada da Costa, 1499-002 Cruz Quebrada, Portugal; mperalta@fmh.ulisboa.pt (M.P.); duarteneto13@gmail.com (D.H.-N.); 2 ISAMB, Faculdade de Medicina, Universidade de Lisboa, 1649-028 Lisboa, Portugal; 3 Escuela de Doctorado, University of Huelva, 21071 Huelva, Spain; diego.gomez@dpee.uhu.es; 4 Champalimaud Clinical Center, Champalimaud Centre for the Unknown, Champalimaud Foundation, 1400-038 Lisbon, Portugal; diana.frasquilho@research.fchampalimaud.org; 5 Departamento de Educação Física e Desporto, Universidade da Madeira, 9020-105 Funchal, Portugal; erubiog@staff.uma.pt; 6 Interactive Technologies Institute, LARSyS, 9020-105 Funchal, Portugal; 7 Department of Social, Developmental and Educational Psychology, University of Huelva, 21007 Huelva, Spain

**Keywords:** active travel, walking, cycling, mental health

## Abstract

Physical activity (PA) is suggested to have a protective effect against depression. One way of engaging in PA is through active commuting. This review summarises the literature regarding the relationship between active commuting and depression among adults and older adults. A systematic review of studies published up to December 2019, performed in accordance with the Preferred Reporting Items for Systematic Reviews and Meta-Analysis guidelines, was conducted using three databases (PubMed, Scopus, and Web of Science). A total of seven articles were identified as relevant. The results from these studies were inconsistent. Only two presented a significant relationship between active commuting and depression symptoms. In those two studies, switching to more active modes of travel and walking long distances were negatively related to the likelihood of developing new depressive symptoms. In the other five studies, no significant association between active travel or active commuting and depression was found. The relationship between active commuting and depression symptoms in adults is not clear. More studies on this topic are necessary in order to understand if active commuting can be used as a public health strategy to tackle mental health issues such as depression.

## 1. Introduction

Depression is a mental health problem that affects more than 300 million adults worldwide [1]. The prevalence of depression has been rising globally, and it was projected to be the second-largest cause for the burden of disease by 2020 [2]. Depression is already the greatest non-communicable disease contributing to the loss of health [1], due to its association with comorbidities [3], risk of suicide [4], and premature mortality [5]. Most cases of depression are treated using pharmacotherapy and psychotherapy [6,7]. However, both types of therapies are expensive and increase health costs for health care systems [8].

Physical activity (PA) is suggested to have a protective effect against depression and a positive effect on the treatment of depression in non-clinical and clinical populations, regardless of sex and age [9,10]. As non-pharmacology therapy, PA does not show drug side effects such as weight gain, insomnia, dry mouth, or withdrawal symptoms [11]. On the contrary, besides the positive benefits on depression, it is also associated with positive public health gains such as physical performance and cardiovascular improvements [11].

PA can be performed in several contexts, including sports activities, occupational context, leisure-time activities, domestic activities, and in active commuting. For some adults, walking or cycling while commuting to work or to other places seems to be important for enhancing their daily levels of PA [12,13], because is likely to be sustained when incorporated into people’s daily routine [14]. As a result, active commuting has been related to health and wellbeing improvement [15,16] and associated with fewer transport-related carbon emissions [17]. Thus, it can be expected that commuting actively might also be related to depression symptoms. However, so far, there is no clear evidence of the relationship between active travel or active commuting and depression symptoms. Therefore, the purpose of this study was to review the literature regarding the relationship between PA in active commuting and depression among adults and older adults.

## 2. Materials and Methods

This systematic review was performed in accordance with the Preferred Reporting Items for Systematic Reviews and Meta-Analysis (PRISMA) guidelines [18].

### 2.1. Inclusion Criteria

Articles that presented a relationship between active travel and active commuting and depression symptoms published in peer-reviewed journals up to December 2019 were eligible for inclusion. Eligibility criteria included the following: (1) cross-sectional, prospective, and cohort studies (study design criterion); (2) outcomes included depression or depressive symptoms (outcome measure criterion); (3) active travel or active commuting (relationship criterion); (4) adults and older adults (participants criterion); (5) articles published in English, Portuguese, or Spanish (language criterion); and (6) articles that did not meet the inclusion criteria or did not include findings related to the inclusion criteria were excluded (exclusion criteria).

### 2.2. Search Strategy

Studies published up to December 2019 were identified by searching in electronic databases. The search was undertaken in the PubMed, Scopus, and Web of Science databases. Articles that assessed the relationship between active travel or active commuting and depression were included in this review. The search was performed using the following combination of terms: travel* OR transport* OR commute* OR cycle OR cycling OR bicycle* OR bike* OR walk* OR/AND depress* OR mental health OR psychological health OR anxiety OR psychological function*. Search terms were defined previously and used in each database to identify articles for review. Two reviewers (A.M. and M.P.) independently screened titles and abstracts to identify studies that met the inclusion criteria. Duplicate articles were removed. Relevant articles were retrieved for a full read. Two authors (A.M. and M.P.) reviewed the full text of potential studies, and decisions to include or exclude studies in the review were made by consensus. Disagreements were solved by consensus and, when necessary, a third reviewer served as a judge (D.H.-N. or D.F.).

### 2.3. Data Extraction and Harmonisation

Based on PRISMA [18], a data extraction form was developed. The following information was extracted from each article: authors’ name and year of publication; study design; country; sample characteristics (number of participants, sex, and age); the instrument for assessing depression symptoms; the instrument for assessing active travel or active commuting; main results; and study quality. The extraction was performed by one author (A.M.), and coding was verified by two authors (M.P. and D.H.-N.).

### 2.4. Study Quality and Risk of Bias

Study methodological quality was assessed using a checklist criteria from the Quality Assessment Tool for Quantitative Studies [19]. The checklist comprises 19 items, assessing eight methodological domains: selection bias, study design, confounders, blinding, data collection methods, withdrawals and dropouts, intervention integrity, and analyses. Each section is classified as strong, moderate, and with weak methodological quality. Then, a global rating is determined based on the scores of each component. Two researchers rated the articles (M.P. and D.F.) in each domain and the studies’ overall quality. Discrepancies were resolved by consensus.

### 2.5. Synthesis of Results

This review analysed the relationship between active travel or active commuting and depression. The details for each study, including design, participant characteristics and sample size, measures, main results, and study quality, are presented in a consistent manner. 

## 3. Results

### 3.1. Search Results

**Figure 1:**
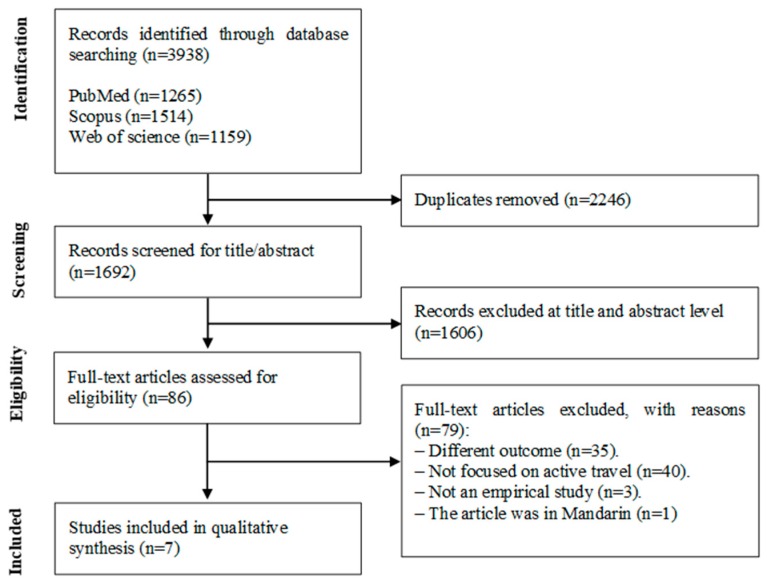
Flow diagram of study selection.

Figure 1 presents the flow citation through the systematic review process. The systematic search yielded 3938 publications. After excluding the duplicates (*n* = 2246), the title and abstract of 1692 articles were screened. After eliminating 1606 articles at the title and abstract level, 86 articles remained and were subsequently read. From the 86 articles, 35 were eliminated for having a different outcome, 40 were eliminated because they were not focused on active travel or active commuting, three were eliminated for not being empirical studies, and one was deleted because it was written in Mandarin. Thus, seven articles were identified as relevant.

### 3.2. Study Characteristics

Table 1 presents the studies’ characteristics. From the seven articles, five had prospective designs and two had cross-sectional designs. In total, all studies combined included 47,300 adults aged over 18 years. Geographically, two were performed in the United States; two in Japan; one in Canada; one in the United Kingdom; and one in several Latin American countries (Argentina, Bolivia, Brazil, Colombia, Ecuador, México, Panamá, Peru, Uruguay, and Venezuela). The Center for Epidemiologic Studies Depression Scale (CES-D) was the most used scale to assess depression symptoms (in five articles). In most articles, the walking distance was self-reported and included leisure time and active commuting. The seven studies were considered of moderate quality.

The older study included in the systematic review was published in 2010, and it examined the effect of walking on incident depressive symptoms in Japanese-American older men with and without chronic disease [20]. The authors used a cohort of men from Hawaii born between 1900 and 1919. Several rounds of examinations were performed; the seventh (which was used in the study) was in 1999–2000. The CES-D 11 scale was used to screen for depressive symptoms, and walking activity was self-reported. It was found that older men, without chronic diseases, in the intermediate (odds ratio (OR) = 0.52, 95% confidence interval (CI): 0.32–0.83) and highest (OR = 0.61, 95% CI: 0.39–0.97) walking groups had significantly lower odds of developing eight-year incident depressive symptoms.

Posteriorly, using a one-year follow-up longitudinal survey, Kai et al. [21] examined the prospective association of walking to work with depressive symptoms among 634 Japanese workers. Depression symptoms were screened by the CES-D scale, and walking activity was self-reported. The authors reported that baseline mean walking to work time was 29.2 ± 18.0 min per day. Walking to work tertiles were calculated; mean values of the low, medium, and high walking to work time tertiles were 13.8 ± 7.0, 29.6 ± 1.9, and 49.7 ± 16.1, respectively. No significant association was found between the depressive symptoms and tertiles of walking to work duration.

In an effort to determine whether walking or depressive symptoms were the stronger predictor of each other, Julien et al. [22] examined the longitudinal associations between walking and depressive symptoms in a population-based sample of 498 Canadian urban-dwelling older adults. Four repeated measures over a five-year period were performed. The geriatric depression scale was used to assess depressive symptoms, and walking was self-reported. Although depressive symptoms predicted walking frequency, walking frequency did not predict depressive symptoms at subsequent time points.

Later, a cross-sectional study based on the National Survey of American Life investigated the odds of having depressive symptoms accordingly to walking frequency in 2978 African-American adults [23]. Walking was measured by self-reported frequency, and depressive symptoms were measured with modified versions of the CES-D scale. In this study, women who reported walking often presented lower odds for depressive symptoms than women who reported never walking (OR = 0.56, 95% CI: 0.38–0.82); however, no significant results were found for men.

In the same year, Kuwahara et al. [24] published a cohort study of 29,082 Japanese workers, examining the prospective associations of physical activity during commuting with the risk of depressive symptoms. During a mean follow-up of 4.7 years, 6177 adults developed depressive symptoms. Physical activity and depressive symptoms (similar to the CES-D scale) were assessed by a self-reported questionnaire. The hazard ratio (HR) for individuals who stand or walk during work was 0.86 (95% CI: 0.81–0.92) compared with sedentary workers. However, walking to and from work was not found to be associated with the risk of depressive symptoms.

More recently, Knott et al. [25] examined whether changes in commute mode were associated with differences in the severity of depressive symptoms in 5474 working adults from the United Kingdom in a population-base prospective cohort study (mean follow-up, 4.65 years). Mode, frequency, and distance of commuting to work were self-reported. The severity of depressive symptoms was assessed using the two-item Patient Health Questionnaire. Baseline asymptomatic adults who altered from inactive to active commuting to work reported less severe symptoms at follow-up than those who remained inactive (β = −0.10, 95% CI: −0.20–0.00). However, among baseline symptomatic adults, longer trips for work were associated with worse symptoms at follow-up (β = 0.64, 95% CI: 0.13–1.16).

The most recent study examined the cross-sectional associations between commute patterns and mental health in 5438 adults using survey data from 11 Latin American cities [26]. Commuting was self-reported, and depression was measured using the CES-D scale. No association was found between non-motorised modes of travel and depression.

## 4. Discussion

This review summarises studies, published up to December 2019, which met the defined criteria. Seven studies were systematically reviewed to address the relationship between active commuting and depression symptoms. In general, although some studies demonstrate that active commuting might be negatively related to depression symptoms, the evidence suggests that the active commuting and depression relationship is unclear.

From the seven studies screened, only two presented a significant relationship between active commuting and depression symptoms. In those two studies [20,25], switching to more active modes of travel and walking long distances were negatively related to the likelihood of developing new depressive symptoms. When examining the effect of walking on incident depressive symptoms in older men, Smith et al. [20] observed that those who walked more had lower rates of prevalent depressive symptoms and that those with chronic diseases who walked longer distances per day were less likely to develop eight-year incident depressive symptoms. Furthermore, the authors suggested that there seems to be a threshold effect in the protective effect of walking on the development of incident depressive symptoms, as few differences were found between the intermediate and high walking groups. This finding is of importance, especially for older adults who have more difficulties in engaging in high volumes of physical activity. More recently, using data from the UK Biobank, a study investigated whether changes in commute mode were associated with differences in the severity of depressive symptoms [25]. It was found that adults who altered from inactive to active commuting to work reported less severe symptoms at follow-up than those who remained inactive. This suggests that commuting behaviours change matters, and thus, active commuting may be used as an intervention strategy to prevent the development of depressive symptoms. Previous investigations in physical activity have shown that, for health purposes, present behaviours may be more important than past behaviours [27]. Active travel offers a suitable way of integrating PA into daily life, and those who use active modes of commuting have higher levels of PA [12,13]. As a result, people who often use more active ways to travel might have better health status [15,16,17]. Hypothetically, it means that active commuting can also have a protective effect on depression symptomology or reduce depression symptoms [9,10]. However, different types of PA have different effects on mental health status.

Among adults, there is evidence that leisure-time PA is more related to mental health problems, and specifically to depression, than other forms of PA [28,29,30]. In five out of seven articles, a significant relationship between active commuting and depression symptoms was not found [21,22,23,24,26]. Cross-sectional evidence from two studies showed that the association between active commuting and depression is not consistent. In one study, performed among Latin American adults, it was found that non-motorised modes of travel were not associated with depression [26]. On the other hand, a study among African-American adults reported that, while women who reported walking often presented a lower probability for depressive symptoms than women who reported never walking, the same pattern was not observed in men [23]. In order to prevent an increase in depressive symptoms, physical activity may need to improve fitness [31]. However, walking in many cases is probably not performed at a high enough intensity to improve fitness. This difference between sexes may be due to a large percentage of African-American women being obese and, thus, having lower exercise capacity [23]. This can lead to walking being a more intense physical activity among African-American women compared to African-American men. The other three studies had a longitudinal design and, in general, concluded that walking was not associated with depressive symptoms at follow-up [21,22,24]. In a one-year follow-up longitudinal survey performed among Japanese workers, it was found that depressive symptoms were not associated with tertiles of walking to work durations [21]. Similarly, in a cohort study with a mean follow-up of 4.7 years also among Japanese workers, walking to and from work was not related to the risk of depressive symptoms [24]. Lastly, a study among Canadian older adults verified that, while depressive symptoms predicted walking frequency at subsequent time points, walking frequency did not predict depressive symptoms [22]. The lack of a significant relationship between active commuting and depression symptoms in these articles are in agreement with previous studies among men and women, which showed that walking distance was not related to depression symptoms [32,33,34]. For those who use an active mode of transportation, transit connectivity matters more for mental health than active travel [26,34]. Furthermore, there is a difference between walking and cycling as a mode of active transportation. Most studies focus on walking as a way of active transportation. Those who usually walk as an active mode of transportation undertake at a lower intensity than cycling [34,35]. This is of particular importance, because PA intensity is a determinant of health. Thus, perhaps in some studies, the level of PA intensity while walking was too low to cause effects on mental health and depression symptoms.

Inconsistent findings identified in this systematic review can be a reflection of the different methodologies used and the different geographical areas and social contexts examined. Measures of active commuting widely varied from study to study, although all were self-reported. Some studies focused on volume [20,21,24], others on frequency [22,23]; one study focused only on the mode of commuting [26] and another one examined the transition from non-active commuting to active commuting [25]. The type of active commuting also differed among studies. Most investigated only walking [20,21,22,23,24], while two studies investigated active commuting without specifying the type [25,26]. Additionally, the context of the commuting was different among studies. Three studies referred to commuting to and from work [21,24,25]. The others did not specify the context of commuting. Due to this, comparability among studies is very limited. Regarding physical activity and active commuting volume and frequency are important information. Results may differ based on these variables. Although some studies examined volume or frequency, they used different time frames. Studies rarely consider both volume and frequency together. Another important aspect for comparability of the results is the type of active commuting, mainly because different types of commuting (e.g., walking versus cycling) have different intensities and volumes associated to them. Future studies should include frequency and volume and be as specific as possible when analysing active commuting. Preferably, similar methods should be used among studies to improve comparability. In addition to the studies being undertaken in different geographic areas and using different ways to measure active transport, it is important to mention that different scales were used to assess symptoms of depression. The use of different scales between the articles may also have contributed to the inconsistency of the results that were observed.

The social and built environment is an important factor to be considered in mental health. The neighbourhood socioeconomic level and built-environment characteristics are associated with a higher probability of screening positively for depression [36]. Walking or cycling in some built-environments can contribute to the development of depression symptoms [26,36]. Furthermore, for some people, active transportation is the only mode of transportation, because they do not have a car or access to mass transit options. In these cases, an active mode of transportation is not an option, it is a mandatory condition. As a result, transport exclusion is associated with social exclusion, which might increment potential mental health problems such as depression [37].

These study review findings should be considered in light of some limitations. Firstly, in all screened articles, the information on commuting and depression was self-reported, which may be subject to bias. Moreover, self-reported PA may have led to overestimates of total PA. Secondly, several articles do not make a clear distinction between active transport as a form of displacement and active transport as a form of leisure (e.g., walking for leisure). This lack of distinction precludes a more rigorous analysis of the relationship between active transport and depression symptoms. Thirdly, some studies were focused on specific populations, such as Japanese-American older men in Hawaii, African-American adults, or workers. This may compromise the generalisability of the results to the general population. Finally, two studies were cross-sectional, which makes it impossible to infer causality. Finally, search terms were selected to identify articles that associate active transportation and depression symptoms. Nevertheless, several articles were subsequently excluded, because neither the title nor abstract contained terms that could be associated with active travel or active commuting.

## 5. Conclusions

This systematic review shows that the relationship between active commuting and depression symptoms in adults and older adults is inconsistent. Overall, out of seven studies, only two found beneficial effects of active commuting on depression symptoms. Active commuting improves daily PA levels, which is known to be related to depression symptoms. Thus, hypothetically, active commuting could be related to depression. However, five studies did not find a significant relationship between active commuting and depression symptoms. More studies on this topic are necessary in order to understand if active commuting can be used as a public health strategy to tackle mental health problems such as depression. Future studies should include frequency and volume and be as specific as possible when analysing active commuting. Preferably, similar methods should be used among studies to improve comparability. Furthermore, future studies should be more specific regarding the context of commuting.

## Figures and Tables

**Table 1:** Characteristics of the studies.

Author, Year	Study Design	Sample and Country	Depression Measure	Active Commuting Measure	Observations	Main Results	Methodological Quality
Smith et al., 2010	Prospective cohort.	*n* = 3196 Japanese-American men who were involved in the Honolulu Heart Program aged 71–93 years; mean age 77 years. United States.	Center for Epidemiologic Studies Depression Scale (CES-D 11)	Participants reported how many city blocks they walked each day. Blocks were converted into miles using 12 blocks per mile as a conversion factor. This assessment was developed from the Harvard Alumni Survey.	Walking distance included leisure-time and active commuting.	Those who walked more had significantly lower rates of prevalent depressive symptoms in cross-sectional analyses. Elderly men, without chronic diseases, who walked longer distances per day were less likely to develop new depressive symptoms over eight years of follow-up.	Moderate
Kai et al., 2011	Prospective (1-year follow-up).	*n* = 634: 536 men, 98 women aged 20–60 years; 36.7 ± 9.2 years. Japan.	Center for Epidemiologic Studies Depression Scale (CES-D)	The duration of leisure-time physical activity and commuting by walking were measured using a self-report questionnaire.	Walking distance included leisure-time physical activity and commuting to work.	The adjusted odds ratio (OR) of depressive symptoms in the highest tertile of leisure-time physical activity was 50% lower (OR = 0.50, 95% CI: 0.26–0.97) than those in the lowest tertile. In contrast, no significant association was found between the risk of depressive symptoms and duration of commuting by walking.	Moderate
Julien et al., 2013	Prospective (5-years follow-up). VoisiNuAge Study.	*n* = 498: 236 men, 262 women aged 68–84 years (74.86 ± 4.18 men and 74.90 ± 3.97 women). Canada.	Geriatric Depression Scale (GDS)	Physical Activity Scale for the Elderly (PASE).	Walking distance included leisure-time and active commuting.	Depressive symptoms predicted walking frequency (higher depressive symptoms were related to fewer walking days), but walking frequency did not predict depressive symptoms at subsequent time points.	Moderate
Torres at el., 2015	Cross-sectional. National Survey of American Life (NSAL).	*n* = 2978: 1903 men, 1075 women aged ≥18 years. United states.	Center for Epidemiologic Studies Depression Scale (CES-D)	Walking was measured with responses to one question from the Americans’ Changing Lives questionnaire.	Walking distance included leisure-time and active commuting.	Women who reported often walking had lower odds for depressive symptoms than women who reported never walking (OR = 0.56, 95% CI: 0.38–0.82). Walking frequency was not related to depressive symptoms in men.	Moderate
Kuwahara et al., 2015	Prospective (5-year follow-up). Japan Epidemiology Collaboration on Occupational Health (J-ECOH) Study.	*n* = 29,082 workers: 24,676 men, 4406 women aged 20–64 years; mean age 42.7 years. Japan.	Epidemiologic Studies Depression Scale (CES-D), Self-Rating Depression Scale (SDS)	Participants were asked whether they regularly engaged in any physical activity during leisure. Duration of walking to and from work was self-reported and categorised as <20minutes, 20–40 min, and ≥ 40 min.	It assessed leisure-time physical activity, physical activity at work, and active commuting.	Leisure exercise showed a U-shaped association with the risk of depressive symptoms. Walking to and from work was not associated with depressive symptoms.	Moderate
Knott et al., 2018	Population-based prospective cohort. UK Biobank.	*n* = 5474, aged 37–73 years. United Kingdom.	Patient Health Questionnaire (PHQ-2)	Participants reported the frequency of trips from home to work (trips/week); the distance travelled (miles); and the mode of transport used (“car or motor vehicle” (hereafter ”car” for simplicity), ”public transport”, and ”walk” and/or ”cycle”).	Paper was focused on modes of travel to work.	Participants who were asymptomatic at baseline and switched to more active modes of commuting tended to report a lower severity of symptoms at follow-up than those who continued to travel inactively (β = −0.10, 95% CI: −0.20–0.00). Among commuters who were symptomatic at baseline, longer journeys were associated with worse symptoms at follow-up (β = 0.64, 95% CI: 0.13–1.16). Shifting from exclusive car use towards more active commuting may help prevent and attenuate depressive symptoms in working adults.	Moderate
Wang et al., 2019	Cross-sectional. La Encuesta CAF 2016.	*n* = 5438. Argentina, Bolivia, Brazil, Colombia, Ecuador, México, Panamá, Peru, Uruguay, and Venezuela.	Center for Epidemiologic Studies Depression Scale (CES-D)	Participants reported commuting time during a “normal day”, uncongested commuting time, and travel mode commonly used for the commute.	Different modes of travel were assessed.	Every 10 more minutes of commuting time is associated with a 0.5% (*p* = 0.011) higher probability of screening positively for depression. There were not found any significant associations between non-motorised modes of travel and depression.	Moderate

## References

[1] WHO (2017). Depression and Other Common Mental Disorders. Global Health Estimates.

[2] Chapman D.P., Perry G.S. (2008). Depression as a major component of public health for older adults. Prev. Chronic Dis..

[3] Vancampfort D., Correll C.U., Galling B., Probst M., De Hert M., Ward P.B., Rosenbaum S., Gaughran F., Lally J., Stubbs B. (2016). Diabetes mellitus in people with schizophrenia, bipolar disorder and major depressive disorder: A systematic review and large scale meta-analysis. World Psychiatry.

[4] Ferrari A.J., Charlson F.J., Norman R.E., Patten S.B., Freedman G., Murray C.J., Vos T., Whiteford H.A. (2013). Burden of depressive disorders by country, sex, age, and year: Findings from the global burden of disease study 2010. PLoS Med..

[5] Walker E.R., McGee R.E., Druss B.G. (2015). Mortality in mental disorders and global disease burden implications: A systematic review and meta-analysis. JAMA Psychiatry.

[6] Karyotaki E., Smit Y., Holdt Henningsen K., Huibers M.J., Robays J., de Beurs D., Cuijpers P. (2016). Combining pharmacotherapy and psychotherapy or monotherapy for major depression? A meta-analysis on the long-term effects. J. Affect. Disord..

[7] Khan A., Faucett J., Lichtenberg P., Kirsch I., Brown W.A. (2012). A systematic review of comparative efficacy of treatments and controls for depression. PLoS ONE.

[8] Olfson M., Amos T.B., Benson C., McRae J., Marcus S.C. (2018). Prospective service use and health care costs of Medicaid beneficiaries with treatment-resistant depression. J. Manag. Care Spec. Pharm..

[9] Schuch F.B., Vancampfort D., Firth J., Rosenbaum S., Ward P.B., Silva E.S., Hallgren M., Ponce De Leon A., Dunn A.L., Deslandes A.C. (2018). Physical activity and incident depression: A meta-analysis of prospective cohort studies. Am. J. Psychiatry.

[10] Rebar A.L., Stanton R., Geard D., Short C., Duncan M.J., Vandelanotte C. (2015). A meta-meta-analysis of the effect of physical activity on depression and anxiety in non-clinical adult populations. Health Psychol. Rev..

[11] Tasci G., Baykara S., Gurok M.G., Atmaca M. (2019). Effect of exercise on therapeutic response in depression treatment. Psychiatry Clin. Psychopharmacol..

[12] Sahlqvist S., Song Y., Ogilvie D. (2012). Is active travel associated with greater physical activity? The contribution of commuting and non-commuting active travel to total physical activity in adults. Prev. Med..

[13] Yang L., Panter J., Griffin S.J., Ogilvie D. (2012). Associations between active commuting and physical activity in working adults: Cross-sectional results from the Commuting and Health in Cambridge study. Prev. Med..

[14] Hillsdon M., Thorogood M. (1996). A systematic review of physical activity promotion strategies. Br. J. Sports Med..

[15] Berglund E., Lytsy P., Westerling R. (2016). Active traveling and its associations with self-rated health, BMI and physical activity: A comparative study in the adult Swedish population. Int. J. Environ. Res. Public Health.

[16] Humphreys D.K., Goodman A., Ogilvie D. (2013). Associations between active commuting and physical and mental wellbeing. Prev. Med..

[17] Chapman R., Keall M., Howden-Chapman P., Grams M., Witten K., Randal E., Woodward A. (2018). A cost benefit analysis of an active travel intervention with health and carbon emission reduction benefits. Int. J. Environ. Res. Public Health.

[18] Moher D., Liberati A., Tetzlaff J., Altman D.G., Group P. (2009). Preferred reporting items for systematic reviews and meta-analyses: The PRISMA statement. Ann. Intern. Med..

[19] National Collaborating Centre for Methods and Tools Quality Assessment Tool for Quantitative Studies Method. http://www.nccmt.ca/resources/search/14.

[20] Smith T.L., Masaki K.H., Fong K., Abbott R.D., Ross G.W., Petrovitch H., Blanchette P.L., White L.R. (2010). Effect of walking distance on 8-year incident depressive symptoms in elderly men with and without chronic disease: The Honolulu-Asia Aging Study. J. Am. Geriatr. Soc..

[21] Kai Y., Nagamatsu T., Yamaguchi Y., Tokushima S. (2011). Effect of leisure-time physical activity and commuting by walking on depressive symptoms among Japanese workers. Bull. Phys. Fit. Res. Inst..

[22] Julien D., Gauvin L., Richard L., Kestens Y., Payette H. (2013). Longitudinal associations between walking frequency and depressive symptoms in older adults: Results from the VoisiNuAge study. J. Am. Geriatr. Soc..

[23] Torres E.R., Sampselle C.M., Neighbors H.W., Ronis D.L., Gretebeck K.A. (2015). Depressive Symptoms and Walking in African-Americans. Public Health Nurs..

[24] Kuwahara K., Honda T., Nakagawa T., Yamamoto S., Akter S., Hayashi T., Mizoue T. (2015). Associations of leisure-time, occupational, and commuting physical activity with risk of depressive symptoms among Japanese workers: A cohort study. Int. J. Behav. Nutr. Phys. Act..

[25] Knott C.S., Panter J., Foley L., Ogilvie D. (2018). Changes in the mode of travel to work and the severity of depressive symptoms: A longitudinal analysis of UK Biobank. Prev. Med..

[26] Wang X., Rodriguez D.A., Sarmiento O.L., Guaje O. (2019). Commute patterns and depression: Evidence from eleven Latin American cities. J. Transp. health.

[27] Marques A., Peralta M., Martins J., de Matos M.G., Brownson R.C. (2017). Cross-sectional and prospective relationship between physical activity and chronic diseases in European older adults. Int. J. Public Health.

[28] Lampinen P., Heikkinen R.L., Ruoppila I. (2000). Changes in intensity of physical exercise as predictors of depressive symptoms among older adults: An eight-year follow-up. Prev. Med..

[29] Siefken K., Junge A., Laemmle L. (2019). How does sport affect mental health? An investigation into the relationship of leisure-time physical activity with depression and anxiety. Hum. Mov..

[30] Joshi S., Mooney S.J., Kennedy G.J., Benjamin E.O., Ompad D., Rundle A.G., Beard J.R., Cerda M. (2016). Beyond METs: Types of physical activity and depression among older adults. Age Ageing.

[31] Dishman R.K., Sui X., Church T.S., Hand G.A., Trivedi M.H., Blair S.N. (2012). Decline in cardiorespiratory fitness and odds of incident depression. Am. J. Prev. Med..

[32] McKercher C.M., Schmidt M.D., Sanderson K.A., Patton G.C., Dwyer T., Venn A.J. (2009). Physical activity and depression in young adults. Am. J. Prev. Med..

[33] Kull M., Ainsaar M., Kiive E., Raudsepp L. (2012). Relationship between low depressiveness and domain specific physical activity in women. Health Care Women Int..

[34] Mytton O.T., Panter J., Ogilvie D. (2016). Longitudinal associations of active commuting with wellbeing and sickness absence. Prev. Med..

[35] Costa S., Ogilvie D., Dalton A., Westgate K., Brage S., Panter J. (2015). Quantifying the physical activity energy expenditure of commuters using a combination of global positioning system and combined heart rate and movement sensors. Prev. Med..

[36] Sampson L., Martins S.S., Yu S., Chiavegatto Filho A.D.P., Andrade L.H., Viana M.C., Medina-Mora M.E., Benjet C., Torres Y., Piazza M. (2019). The relationship between neighborhood-level socioeconomic characteristics and individual mental disorders in five cities in Latin America: Multilevel models from the World Mental Health Surveys. Soc. Psychiatry Psychiatr. Epidemiol..

[37] Lucas K. (2012). Transport and social exclusion: Where are we now?. Transp. Policy.

